# Age-Related Yield of Adipose-Derived Stem Cells Bearing the Low-Affinity Nerve Growth Factor Receptor

**DOI:** 10.1155/2013/372164

**Published:** 2013-11-24

**Authors:** Raquel Cuevas-Diaz Duran, Maria Teresa González-Garza, Alejandro Cardenas-Lopez, Luis Chavez-Castilla, Delia Elva Cruz-Vega, Jorge E. Moreno-Cuevas

**Affiliations:** ^1^Cell Therapy Department, School of Medicine, Tecnologico de Monterrey, Avenue Morones Prieto 3000 Pte., 64710 Monterrey, \ NL, Mexico; ^2^Neomedic Plastic Surgery Center, Rio Guadalquivir 301 Ote, San Pedro Garza Garcia, 66220 Monterrey, NL, Mexico

## Abstract

Adipose-derived stem cells (ADSCs) are a heterogeneous cell population that may be enriched by positive selection with antibodies against the low-affinity nerve growth factor receptor (LNGFR or CD271), yielding a selective cell universe with higher proliferation and differentiation potential. This paper addresses the need for determining the quantity of ADSCs positive for the CD271 receptor and its correlation with donor's age. Mononuclear cells were harvested from the lower backs of 35 female donors and purified using magnetic beads. Multipotency capacity was tested by the expression of stemness genes and through differentiation into preosteoblasts and adipocytes. A significant statistical difference was found in CD271^+^ concentrations between defined age intervals. The highest yield was found within women on the 30–40-year-old age range. CD271^+^ ADSCs from all age groups showed differentiation capabilities as well as expression of typical multipotent stem cell genes. Our data suggest that the amount of CD271^+^ cells correlates inversely with age. However, the ability to obtain these cells was maintained through all age ranges with a yield higher than what has been reported from bone marrow. Our findings propose CD271^+^ ADSCs as the primary choice for tissue regeneration and autologous stem cell therapies in older subjects.

## 1. Introduction

It has been demonstrated that adipose tissue represents an abundant source of mesenchymal stem cells as well as those obtained from bone marrow. Furthermore, adipose-derived stem cells (ADSCs) have similar differentiation capability, morphology, and phenotype as mesenchymal stem cells collected from umbilical cord blood or bone marrow [1–5]. 

ADSCs, like bone marrow derived stem cells, adhere to plastic producing fibroblast-like colonies, have a high proliferative capacity, express common surface antigens, and can differentiate *in vitro* and *in vivo* toward cells of mesodermal lineage [6]. Also, ADSCs have the ability to be induced into cells derived from all three germ layers [6–10] and are capable of suppressing immunoreactivity [11], making them ideal for stem cell-based therapies.

A typical ADSCs extraction protocol yields heterogeneous cell populations which may be homogenized through culture. However, time and culture conditions may cause changes in their phenotype due to sequential differences in antigen expression [12]. A homogeneous, fully characterized ADSCs population is desirable for use in clinical applications, an event that can be reached using antibodies [13].

Different cell surface receptors, such as the low-affinity nerve growth factor receptor (CD271), have been used as targets for ADSCs antibody based isolation. This cell surface marker defines a mesenchymal stem cell (MSC) subpopulation and has been used for the enrichment of cells collected from bone marrow aspirates [13, 14] and lipoaspirates [15]. Jones and McGonagle [16] demonstrated that the CD271 antigen is one of the most selective markers for enriching MSC from human bone marrow. Bone marrow MSC positive for CD271 antigen have a 10- to 1000-fold higher proliferative capacity when compared to MSC isolated by plastic adherence [13] and have both immunosuppressive and lymphohematopoietic engraftment-promoting properties [17]. Similarly, CD271^+^ cells immunomagnetically selected from ADSCs showed a higher clonogenic and differentiation potential compared to plastic adherent ADSCs [15]. Additionally, Yamamoto et al. [18] isolated CD271^+^ cells from mouse subcutaneous adipose tissue and demonstrated that their differentiation capability into adipocytes, osteoblasts, and neuronal cells was higher when compared to plastic adherent ADSCs. 

These findings suggest that CD271^+^ ADSCs are an excellent homogeneous subset of stem cells for clinical applications. This study's aim is to determine if there is a relationship between donor's age and CD271^+^ cell yield in freshly isolated ADSCs. Also, stem cell gene expression in CD271^+^ ADSCs will be addressed in order to verify their multipotency. 

## 2. Material and Methods

### 2.1. Patients and Tissue Sampling

Thirty-five female healthy patients aged between 30 and 65 years undergoing cosmetic liposuction at NeoMedics (Monterrey, Nuevo Leon, Mexico) participated in our study. Five samples were used for verifying previous reports where areas of higher cellular densities were determined, and the rest were used for age correlation assessment. Adipose tissue from this group was obtained from inner thigh, trochanteric region, lower back, and abdomen, in order to corroborate the best area for sample collection. Exclusion criteria included diabetes mellitus, allergies, previous liposuctions, medication, or vitamin intake two weeks before surgery, hematologic disorders, lipodystrophies, morbid obesity, and chronic use of corticoids. For age correlation studies, 120 mL of adipose tissue was harvested from 30 donors within the following age periods: 30–40 (*n* = 10), 41–50 (*n* = 10), and 51–65 (*n* = 10). Donors' body mass indexes were within the same range (mean = 23, standard deviation = 1.58). Lipoaspirates were obtained under written informed consent and all procedures were performed by the same plastic surgeon using the tumescent technique. This technique consists of infiltration of the fat compartment with a large volume of a highly diluted local anesthetic and epinephrine solution for induction of vasoconstriction. Under general anesthesia, patients were infiltrated locally with Klein solution, consisting of 0.1% lidocaine, 10 mg of sodium bicarbonate, and epinephrine diluted 1 : 1,000,000 in 1000 mL sterile physiologic normal saline. Liposuction was performed using a vacuum system connected to blunt-ended cannulas (Mentor, California, USA) with a diameter of 2.1 mm and a length of 15 cm. Fat was removed 10 min after infiltration.

Cell density determination and characterization studies were performed with samples obtained from abdomen, lower back, inner thigh, and trochanteric regions. 

### 2.2. Cell Isolation and Culture

ADSC isolation was performed as previously described by Zuk et al. [19]. Briefly, aspirated tissue was washed at least 3 times with phosphate-buffered saline (PBS, Sigma-Aldrich, St. Louis, MO, USA) and 1% penicillin and streptomycin (Sigma-Aldrich). The pellet obtained from the first wash was collected and processed as described by Francis et al. [20]. Washed lipoaspirate was digested with 0.1% collagenase type I-S (Sigma-Aldrich) dissolved in Hank's buffer (Gibco, Grand Island, NY, USA). Digestion took place in a shaker at 37°C and 250 rpm during 60 min. The enzyme was inactivated by adding an equal volume of Dulbecco's Modified Eagle's medium (DMEM-F12, Gibco) supplemented with 10% fetal bovine serum (FBS, Gibco). Mature adipocytes and connective tissue were separated from pellets of mononuclear cells by centrifugation at 800 g for 10 min. Debris and dissociated tissue present in pellets were eliminated through filtration using a 100 *μ*m cell strainer and washing with PBS. All pellets were mixed and plated on a Ficoll-Paque (GE Healthcare Bio-Sciences, Piscataway, NJ, USA) gradient to reduce erythrocyte contamination. The collected mononuclear fraction was further washed with PBS and filtered through a 40 *μ*m cell strainer to remove debris and cell clumps. 

 CD271^+^ cells were isolated using magnetic labeling beads bearing an anti-CD271 antibody (MicroBead kit, Miltenyi Biotec, Gladbach, Germany). Cells retained on the column (CD271^+^) and unlabeled cells (CD271^−^) were counted using a haemocytometer and samples of 3 × 10^5^ cells were cultured in 25 cm^2^ flasks with DMEM-F12 supplemented with 10% FBS, 50 U/mL penicillin, and 50 *μ*g/mL streptomycin.

Cells were maintained at 37°C and 5.0% CO_2_ in humidified incubators. Flasks were washed with PBS and media were changed completely every 3-4 days until reaching 70% confluence. Maintenance medium consisted of DMEM-F12 supplemented with 5% FBS, 50 U/mL penicillin, and 50 *μ*g/mL streptomycin. Cells were detached with 0.25% Trypsin-EDTA (Gibco) and centrifuged at 375 g for 10 min. Trypsin-EDTA was inactivated using DMEM-F12 supplemented with 20% FBS. The cell pellet was resuspended with maintenance medium; cells were quantified and plated on 12- and 24-well microplates for RNA extraction and differentiation assays, respectively, or used directly for flow cytometry.

### 2.3. Flow Cytometry

To analyze the expression of specific surface proteins, one sample of 3 × 10^5^ CD271^+^ ADSCs (passage 1) from each age range was incubated for 30 min with 20 *μ*L of blocking reagent and 10 *μ*L of the following fluorochrome conjugated monoclonal antibodies in appropriate combinations: CD34-PE, CD45-FITC, CD90-FITC, CD105-PE, and CD271-APC (BD Biosciences, San Diego, CA, USA). After staining, cells were washed with MACS-BSA stock solution (Miltenyi Biotec, Teterow, Germany), centrifuged, and resuspended in MACS-BSA. Samples were evaluated by the FACSCanto II Instrument and data was analyzed using the FACSDiva software (BD Biosciences). 

### 2.4. Reverse-Transcription Polymerase Chain Reaction

Samples from three donors of each age range were used for gene analysis. Total cellular RNA was isolated from 1.2 × 10^5^ cells using the GenElute Mammalian Total RNA MiniPrep Kit (Sigma). Oligo (dT)-primed reverse transcription was performed using aliquots of 0.5 *μ*g of RNA as templates. Amplification was performed for the genes Sox2, Notch1, Rex1, Oct4, Nanog, and Nestin. The housekeeping gene GAPDH was used as the assay basal amplification control. The primers and annealing temperatures used are listed in Table 1. Products were visualized by electrophoresis on 1.5% agarose gels and images were acquired through a UV transilluminator (DigiDoc-IT, Cambridge, UK). 

### 2.5. Induced Differentiation

To verify differentiation potential, CD271^+^ ADSCs were plated at passage 1 into 24-well microplates over poly-L-lysine (Sigma-Aldrich) coated coverslips at a density of 3 × 10^4^ cells/well. Cells were induced into preosteoblasts and adipocytes for a period of 21 days. Induction media were changed every 3 days.

### 2.6. Osteogenic Differentiation

The osteogenic medium consisted of 2 mM *β*-glycerophosphate (Sigma-Aldrich), 1 nM dexamethasone (Sigma-Aldrich), and 50 *μ*M ascorbate-2-phosphate (Sigma-Aldrich) in *α*-MEM Basal Medium (Gibco). For fixing, cells were washed with PBS and incubated in ice cold 70% ethanol for 1 hour at room temperature. Cells were rinsed with water, stained with Alizarin Red Solution (40 mM, pH 4.1, Sigma-Aldrich) for 30 min, and washed with bidistilled water. Extracellular calcium mineralization was observed under a microscope (AxioImager Z1 fluorescent microscope). 

### 2.7. Adipogenic Differentiation

Adipogenic differentiation was induced with 1 *μ*M dexamethasone, 200 *μ*M indomethacin (Sigma-Aldrich), 1 mM isobutylmethylxanthine (Sigma-Aldrich), and 10 *μ*M insulin (Sigma-Aldrich) in *α*-MEM Basal Medium. For fixing, cells were washed with cold PBS and incubated with 10% formalin for 30 min at room temperature. Cells were air-dried before staining, washed with PBS, and incubated for 60 min with 0.5% Oil Red O (Hycel, Jalisco, Mexico). To avoid nonspecific staining, samples were rinsed several times with PBS.

### 2.8. Statistical Analysis

Cell concentration distributions were analyzed through means and standard deviations. Model fit was tested by analysis of residues and the distribution's normality was determined using the Shapiro-Wilk test and the Kolmogorov-Smirnov goodness of fit test. A single factor ANOVA was used to analyze equality of means between groups. Group means were compared using the Tukey-Kramer method. All analysis and graphs were developed using the R programming language [21].

## 3. Results

### 3.1. Adipose-Derived Stem Cell Yield

Mean mononuclear cell concentrations per mL of starting adipose tissue for each harvest site are shown in Table 2. A significant difference in cell counts (*P* value < 0.0008) was found between lower back and inner thigh donor sites. Lower back and abdomen were the sites with higher mononuclear cell counts and there is no significant difference between their mean values (*P* value = 0.857). The difference between mean mononuclear cells counts from lower back and trochanteric is statistically significant (*P* value < 0.05).

To determine the proportion of ADSCs positive for the CD271 receptor and its relationship with age, lipoaspirate samples from the lower back of 10 female patients from each age range were processed. CD271^+^ ADSCs were isolated and counted. Proportions of CD271^+^ to CD271^−^ ADSCs for each age range were calculated and the results are listed in Table 3. Through a single factor ANOVA analysis, mean CD271^+^ cell counts differ between age groups with a *P* value < 0.001.

**Figure 1:**
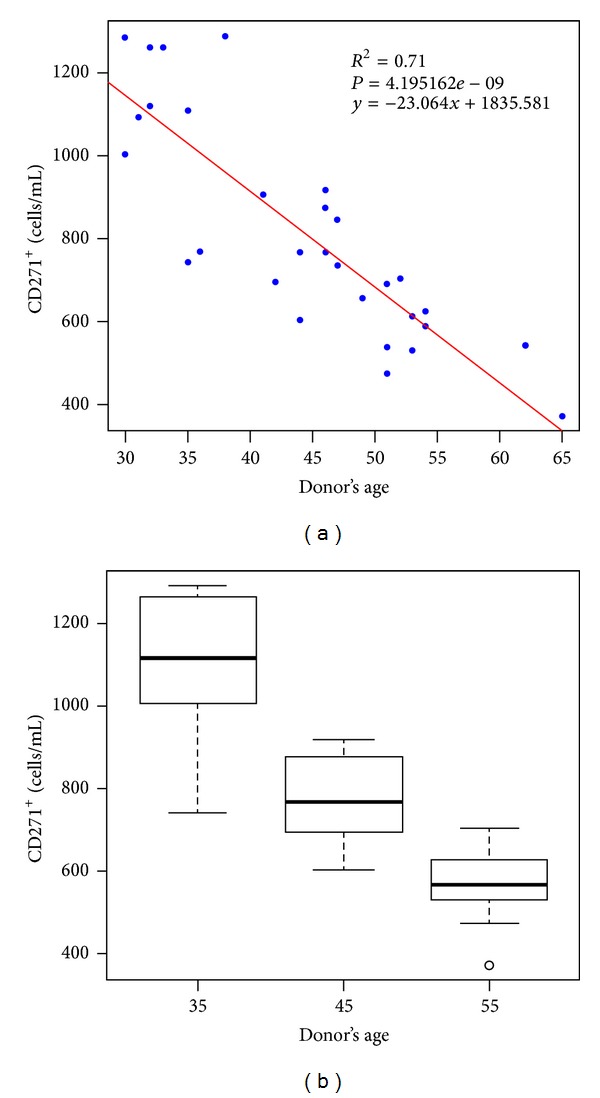
Frequency of CD271^+^ ADSCs per mL of initial adipose tissue taken from the lower backs of 30 healthy female donors with similar body mass index. (a) Distribution of CD271^+^ stem cell/mL of initial adipose tissue and donor's age. Data appear normal and a straight line is adjusted with a goodness of fit of *R* ^2^ = 0.71. The fitted regression equation and its *P* value are shown. (b) Box and whisker plots representing the median, lower, and upper quartiles of CD271^+^ ADSC yield/mL of processed adipose tissue for each age range.

The box and whisker plots describing the distribution of the CD271^+^ ADSC concentration for each age range are depicted in Figure 1(b). Each box plot shows the median CD271^+^ ADSC count calculated for each age group as well as the lower and upper quartiles. The 30–40-year-old range had the highest CD271^+^ ADSC concentration and was significantly different to the amounts obtained from the 41–50-year-old range with a *P* value = 0.01 and to the age range 51–65 with a *P* value < 0.001 as calculated with the Tukey-Kramer test. The difference between the CD271^+^ cell counts between the age ranges 41–50 and 51–65 was also significant with a *P* value = 0.00845. With respect to the analysis of variance model, data appeared normal and adjusted to a straight line with a goodness of fit of *R* ^2^ = 0.71 (Figure 1(a)). Normality was verified by the Kolmogorov-Smirnov test (*D* = 1) and the Shapiro-Wilk test (*P* = 0.078). 

### 3.2. Surface Protein Expression by Flow Cytometry Analysis

**Figure 2:**
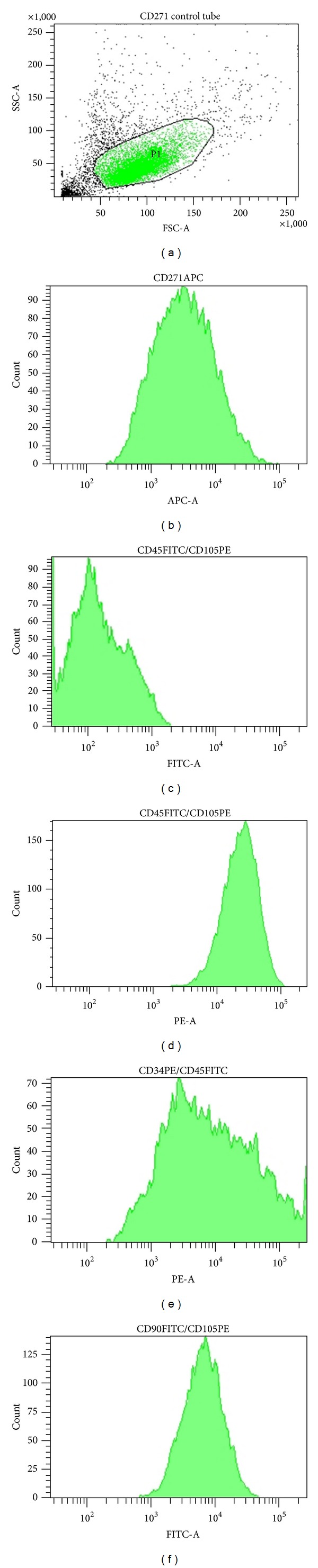
Representative results of flow cytometry analysis are shown for samples of 3 × 10^5^ CD271^+^ ADSCs passage 1 from a 42-year-old donor. (a) The CD271^+^ ADSC population indicated with gage P1 comprises 75.4% of the 10,000 events analyzed. (b) Histogram showing that 73% of cells from gage P1 yield positive for the CD271-APC receptor. (c) Histogram depicting that the CD271^+^ ADSC population is negative for CD45-FITC receptor. (d) Histogram demonstrating that the CD271^+^ ADSC population is positive for the CD105-PE receptor. (e) The histogram shows that 87% of the CD271^+^ ADSC population is positive for the CD34-PE. (f) Histogram demonstrating that 97% of the CD271^+^ ADSC population yields positive for the CD90-FITC receptor.

More than 90% of cultured CD271^+^ ADSCs from all age ranges were positive for stem cell markers CD90 and CD105. There was no expression of the hematopoietic marker CD45. Approximately 70% of CD271^+^ passage 1 ADSC yielded positive for CD271, indicating that this marker is diminished in culture. The expression of CD34 is variable with a mean value of 85.2%. The percentage of mean maker incidence is shown in Table 4. Flow cytometric analysis histograms for cell surface marker expressions are shown in Figure 2.

### 3.3. Multidifferentiation Potential

**Figure 3:**
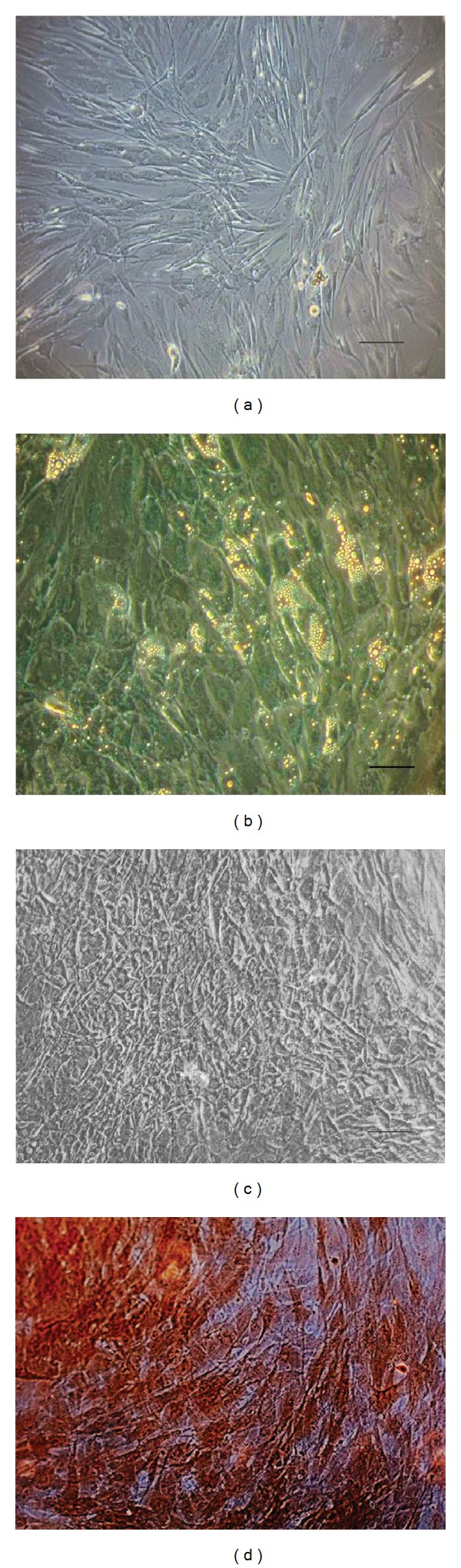
Morphological changes through different induction media. (a) CD271^+^ ADSCs passage 1 after 21 days in culture without induction (20x). (b) Morphological changes visible at day 21 of adipogenic differentiation (20x). (c) Osteogenic differentiation observed at induction day 21 (20x). (d) Microphotograph showing extracellular mineralization unveiled through Alizarin Red staining in CD271^+^ ADSCs after 21 days of osteogenic induction (20x).

The differentiation potential of CD271^+^ ADSCs was tested using the induction media for 21 days. Morphological changes with respect to a control without induction are shown in Figure 3. Cells induced into adipocytes showed intracellular lipid filled vacuoles and exhibited an expanded volume (Figure 3(b)). The amount and size of intracellular lipid vacuoles increased with time. After 21 days of incubation in adipogenic medium, Oil Red staining was used to unveil lipid droplets in red color. Cells incubated with osteogenic induction media appeared polygonal in shape (Figure 3(c)). Extracellular calcium deposition was demonstrated at day 21 by Alizarin Red staining (Figure 3(d)). Control cells without induction lacked red stain. 

### 3.4. Transcription Factor Expression

With respect to genotypes, CD271^+^ ADSCs expressed mRNA for the stem cell genes Sox2, Notch1, Rex1, Oct4, Nanog, and Nestin. 

**Figure 4:**
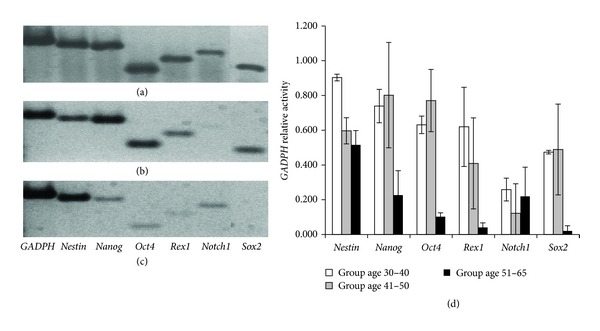
Gene expression analysis. The upper image shows a representative agarose gel electrophoresis showing the expression of selected stem cell genes from the following donor age groups: (a) 30–40 years old, (b) 41–50 years old, and (c) 51–65 years old; (d) shows the average of the GADPH relative gene expression and the error bars indicate the standard deviation in samples from the same age group.

Although the selected stem cell genes were expressed on the samples from the three groups, there was a variation between the groups and among subjects of the same group. Variations were not significant in patients from 30 to 50 years old; however, on subjects from the older group, there was a decrease on the expression of all the analyzed genes. A representative gel image of the RT-PCR products from the three groups is shown in Figure 4. 

## 4. Discussion

Adult stem cells can be isolated from various tissue sources, bone marrow being the most widely used in clinical applications. The common problem in isolating adult stem cells is the low yield and the limited amount of tissue to harvest from. Recently, researchers found that the frequency of stem cells within adipose tissue is approximately 8-fold higher than in bone marrow [22]. Our results demonstrated that lower back and abdomen were the sites with the highest mononuclear cell concentrations, in agreement with the results reported by Padoin et al. [23].

Cells harvested from the stromal vascular fraction comprise a heterogeneous population including preadipocytes, adipocytes, pericytes, endothelial cells, ADSCs, fibroblasts, monocytes, macrophages, and lymphocytes [10], responding differently to stimuli. For the above, it is important to reduce cell heterogeneity before the use of adipose-derived stem cells for regenerative therapies. Exposing a heterogeneous population of stem cells to an induction medium might result in subpopulations undergoing differentiation into different lineages, which could decrease its effectiveness. Therefore, the use of monoclonal antibodies has been proposed for stem cell isolation and selection, in order to decrease this heterogeneity. 

Recently, CD271 (LNGFR) has been described as the optimal selective marker for the purification and isolation of ADSCs [15] and bone marrow derived stem cells [13, 14]. Cells bearing this receptor have a higher multipotency and proliferative capability when compared to the whole population of unselected ADSCs. Studies performed at our laboratory have demonstrated that the expression of CD271 decreases with culture time, perhaps induced by plastic adherence and culture conditions. Such results are in agreement with what was found for bone marrow derived stem cells leading Quirici et al. [13] to the hypothesis that CD271 might be a marker for resting primitive MSC. 

Cultured CD271^+^ ADSCs from all age groups studied were positive for stem cell markers Thy1 (CD90) and endoglin (CD105). No expression was found for the hematopoietic marker CD45. Positive expression of CD90 and CD105, as well as negative expression of CD45, is some of the minimal criteria for stem cells definition proposed by the Mesenchymal and Tissue Stem Cell Committee of the International Society for Cellular Therapy [24]. Our results showed that CD271^+^ ADSCs samples from all groups, including those from older patients, express the core transcription factors associated with cell self-renewal: Sox2, Oct4, and Nanog [25–29]. Similarly, CD271^+^ ADSCs samples expressed Rex1/ZFP42, a known marker of pluripotency [30] and Notch1, a receptor of the Notch signaling pathway which plays a role in stem cell self-renewal and differentiation [31]. CD271^+^ ADSCs highly expressed Nestin, a gene responsible for synthesizing nestin, an intermediate cytoskeletal protein, which has been related with proliferative cells in which speedy cytoskeleton organization is vital [32]. A reduction in these genes' expression was seen in donors from the older age range suggesting that in older subjects, the stemness gene expressions decrease, however, remain active, and give the possibility to respond to any induction. Nevertheless, it is important to perform several induction protocols, in order to establish its potential capability. 

Studying CD271^+^ ADSCs is important for elucidating the relationship between the CD271 receptor with multipotency and proliferation, as compared to CD271^−^ ADSCs. Since CD271^+^ ADSCs are a subset of mononuclear cells, it is safe to correlate CD271^+^ concentrations with the size of mononuclear cell populations. We found the highest concentration of mononuclear cells in the patients in the range of 30–40 years as well as CD271^+^ ADSCs.

The cell distribution behaved similarly, decreasing both with age. Our results show that there is a significant statistical difference between the CD271^+^ ADSCs concentrations for each age group range, with the 30–40-year-old range containing the highest cell concentration. Data appeared normal and a straight line was fitted indicating that there is a correlation between the CD271^+^ ADSCs concentration and age. We attribute this correlation to a decrease in stem cell number. This finding is similar to what was found by Yamada et al. [33], who concluded that the number of ADSCs positive for CD271 per adipose tissue weight decreased in aged mice.

The proportion of cells bearing the CD271 receptor, as compared to mononuclear cells, decreased by more than 30% from the youngest age group (30–40) to the oldest group (51–65). Such a decrease is less than what was found by Stolzing et al. [34], who determined that the number of colony forming unit fibroblasts (CFU-F's) formed by bone marrow MSC declined by more than 50% from the youngest to the aged subjects.

The frequency of CD271^+^ ADSCs found per mL of lipoaspirate is higher than what has been reported in bone marrow aspirates. The average lowest proportion of CD271^+^ to CD271^−^ ADSCs obtained in our study was 1.8% while the relation of CD271^+^ MSC from bone marrow was 0.34% in Quirici et al. [13], 0.28% in Cox et al. [35], and 0.0017–0.0201% in Alvarez-Viejo et al. studies [36]. In this sense a hallmark of our study is that, regarding frequency, CD271^+^ ADSCs are a better source of donor cells for stem cell-based therapies and tissue engineering.

In conclusion, our results suggest that the proportion of CD271^+^ ADSCs isolated from lower back decreases with age; however, cells positive for this marker were present in all our age groups and their frequency was higher than what has been found in bone marrow. These findings strongly propose CD271^+^ ADSCs homogeneous subpopulations as the primary collection choice for tissue regeneration and for autologous stem cell therapies in older subjects. 

## Figures and Tables

**Table 1:** Primer sets and annealing temperatures used for reverse transcription PCR.

Gene	Primer sequence (5′-3′)	Annealing temperature
Sox2		
(fwd) (rev)	ACTTTTGTCGGAGACGGAGA GTTCATGTGCGCGTAACTGT	55°C
Notch1		
(fwd) (rev)	TCACGCTGACGGAGTACAAG CCACACTCGTTGACATCCTG	55°C
Rex1		
(fwd) (rev)	GGCGGAAATAGAACCTGTCA CTTCCAGGATGGGTTGAGAA	55°C
Oct4		
(fwd) (rev)	AGTGAGAGGCAACCTGGAGA ACACTCGGACCACATCCTTC	55°C
Nanog		
(fwd) (rev)	TTCCTTCCTCCATGGATCTG TCTGCTGGAGGCTGAGGTAT	55°C
Nestin		
(fwd) (rev)	AACAGCGACGGAGGTCTCTA TTCTCTTGTCCCGCAGACTT	60°C
GAPDH		
(fwd) (rev)	GAGTCAACGGATTTGGTCGT TTGATTTTGGAGGGATCTCG	60°C

Sox2: SRY (sex determining region Y)-box 2; Notch1: Notch homolog 1; Oct4: octamer-binding transcription factor 4; GAPDH: glyceraldehyde 3-phosphate dehydrogenase.

**Table 2:** Data gathered from 5 female patients 30–40 years old. Data expressed in mean mononuclear cells/mL of initial fat sample.

Donor site	Mononuclear cells/mL
Inner thigh	13,062.5 ± 5385
Trochanteric	21,333 ± 6308
Lower back	40,000 ± 9720
Abdomen	38,500 ± 8631

**Table 3:** Data gathered from 10 female patients per age range. Samples were obtained from lower back. Data is expressed as mean ± standard deviation of CD271^−^ and CD271^+^ cells/mL of initial fat sample.

Donor's age	CD271^−^ cells/mL	CD271^+^ cells/mL	CD271^+^/CD271^−^
30–40	34,189 ± 8190	991 ± 205	2.89%
41–50	33,733 ± 5100	789 ± 104	2.33%
51–65	32,619 ± 4607	595 ± 146	1.82%

**Table 4:** Mean expression of cell surface CD markers of CD271^+^ ADSCs passage one.

Antibody	Conjugate	Mean CD271^+^ cells/mL
CD271^+^	APC	70.9%
CD90^+^/CD105^+^	FITC/PE	99%
CD45^−^/CD105^+^	FITC/PE	98.5%
CD45^−^	FITC	99.6%
CD34^+^	PE	85.2%

## Abbreviations

ADSCs: Adipose-derived stem cells
LNGFR, CD271: Low-affinity nerve growth factor receptor
MSC: Mesenchymal stem cell.
